# Different effects of LDH-A inhibition by oxamate in non-small cell lung cancer cells

**DOI:** 10.18632/oncotarget.2620

**Published:** 2014-10-22

**Authors:** Yang Yang, Dan Su, Lin Zhao, Dan Zhang, Jiaying Xu, Jianmei Wan, Saijun Fan, Ming Chen

**Affiliations:** ^1^ Department of Radiation Therapy, Zhejiang Cancer Hospital, Hangzhou, China; Zhejiang Key Laboratory of Radiation Oncology, Hangzhou, China; ^2^ School of Radiation Medicine and Protection, Medical College of Soochow University, Suzhou, China; ^3^ Cancer Research Institute, Zhejiang Cancer Hospital, Hangzhou, China; ^4^ Tianjin Key Laboratory of Molecular Nuclear Medicine, Institute of Radiation Medicine, Chinese Academy of Medical Sciences and Peking Union Medical College, Tianjin, China

**Keywords:** lactate dehydrogenase A, Warburg effect, G0/G1 arrest, autophagy, apoptosis, Akt/mTOR

## Abstract

Higher rate of glycolysis has been long observed in cancer cells, as a vital enzyme in glycolysis, lactate dehydrogenase A (LDH-A) has been shown with great potential as an anti-cancer target. Accumulating evidence indicates that inhibition of LDH-A induces apoptosis mediated by oxidative stress in cancer cells. To date, it's still unclear that whether autophagy can be induced by LDH-A inhibition. Here, we investigated the effects of oxamate, one classic inhibitor of LDH-A in non-small cell lung cancer (NSCLC) cells as well as normal lung epithelial cells. The results showed that oxamate significantly suppressed the proliferation of NSCLC cells, while it exerted a much lower toxicity in normal cells. As previous studies reported, LDH-A inhibition resulted in ATP reduction and ROS (reactive oxygen species) burst in cancer cells, which lead to apoptosis and G_2_/M arrest in H1395 cells. However, when being exposed to oxamate, A549 cells underwent autophagy as a protective mechanism against apoptosis. Furthermore, we found evidence that LDH-A inhibition induced G_0_/G_1_ arrest dependent on the activation of GSK-3β in A549 cells. Taken together, our results provide useful clues for targeting LDH-A in NSCLC treatment and shed light on the discovery of molecular predictors for the sensitivity of LDH-A inhibitors.

## INTRODUCTION

Lung cancer is one of the most common cancers and causes more than 1.37 million deaths worldwide. The incidence of lung cancer is still on the rise, due to the prevalence of smoking and air pollution, especially in the developing countries [1]. In spite of recent progress in the treatment of lung cancer, including TKIs (tyrosine kinase inhibitors) and anti-VEGF, the prognosis of lung cancer remains poor, with 5-year survival rate approximately 18%[2]. Hence, there is an urgent need to develop novel strategies to treat lung cancer.

It is noticed long before that cancer cells have higher uptake of glucose and more dependent on the anerobic glycolysis to produce ATP, the phenomenon is also known as “Warburg effect” [3]. In recent years, targeting energy metabolism has returned to the battlefield of fighting against cancer, more details and molecular mechanisms involved in the “Warburg effect” are increasingly discovered, which not only make us better understand the characteristics of cancer cells, but also provide the Achilles' heel to kill them [4].

Among the numerous enzymes participating in the glycolysis, lactate dehydrogenase A (LDH-A), an isoform of lactate dehydrogenase, is undoubtedly an remarkable anti-cancer target with great developable potential [5, 6]. After the year of 2000, many studies found that LDH-A was abnormally expressed in cancer cells and associated with poor prognosis, suggesting that LDH-A played an important role in tumor maintenance [7-12]. Then, Le, *et al*. reported that inhibition of LDH-A reduced ATP levels and induced apoptosis though the accumulation of reactive oxygen species (ROS) in lymphoma cells [13]. Subsequently, a number of studies found similar results in other types of tumor cells including renal cancer [14], breast cancer [15], hepatocellular carcinoma [16, 17], nasopharyngeal carcinoma [18] and pancreatic cancer [19]. Besides, the results also indicate that LDH-A inhibition could suppress the migration of cancer cells and enhance their sensitivity to the traditional chemotherapy and radiotherapy [18-21]. Especially, the findings are also encouraging in lung cancer cells. Very recently, it was demonstrated that LDH-A was essential for cancer-initiating cell proliferation and could be a feasible therapeutic target for non-small cell carcinoma (NSCLC) treatment in mouse models [22].

Since lung cancer, particularly non-small cell carcinoma is one kind of highly heterogeneous tumors with various genetic expressions, and specific treatment is not always effective to all types of lung cancer [23], we built the hypothesis that different NSCLC cells might exhibit different responses to LDH-A inhibitors. In the present study, we investigated the effect of oxamate, one classic inhibitor of LDH-A [24, 25], in several cell lines of NSCLC, as well as normal lung epithelial HBE cells. The purpose of our study is to examine the effectivity of LDH-A inhibition in NSCLC cells and explore the related mechanism.

## RESULTS

### Different growth inhibition effects of oxamate in NSCLC cells

**Figure 1:**
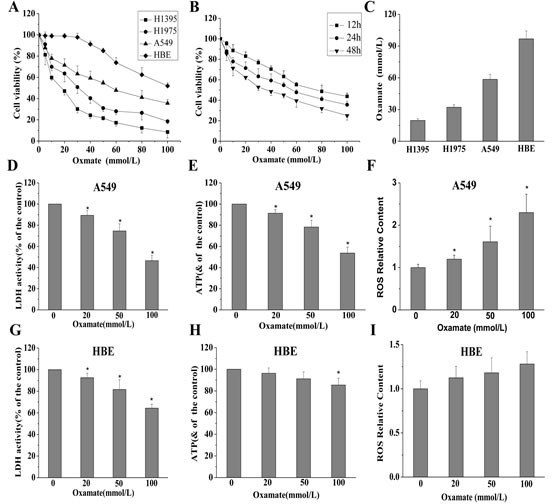
Different effects of oxamate on the cell viability and energy metabolism in NSCLC cells and normal lung epithelial cells (A) A549, H1395, H1975 and HBE cells were exposed to varying doses of oxamate for 24 h, and tested by MTT assay. (B)The effect of oxamate on A549 cells was determined at different time points. (C) IC_50_ of different cells at 24h were calculated by the growth curves. (D-F) A549 cells were treated with 0, 20, 50,100 mM oxamate for 24h, then LDH enzyme activity, ATP and ROS content were determined by commercial kits, respectively. (G-I) The levels of LDH enzyme activity, ATP and ROS content were also assayed in HBE cells. All Data above represented the average of three independent experiments and were shown as means ± SD, * p<0.05 versus control.

Firstly, MTT assays were performed to investigate the effect of LDH-A inhibition by oxamate on the cell proliferation in NCSLC cells and normal lung epithelial cells. As shown in Figure 1A, we found that oxamate obviously inhibited the viability of A549, H1975, H1395 cells in a dose- and time-dependent manner, the IC_50_ (50% inhibitory concentration) at 24h of oxamate sodium were 58.53±4.74, 32.13±2.50 and 19.67±1.53 mmol/L for A549, H1975 and H1395 cells, respectively, while oxamate exerted much lower toxicity in normal lung epithelial cell line HBE cells with its IC_50_ 96.73±7.60 mmol/L.

Then, to further confirm the LDH-A inhibition effect of oxamate as previously reported and understand the alteration of energy metabolism in cells, several intracellular biochemical indicators were detected in both A549 and HBE cells. As a result, after treatment with oxamate for 24h, LDH activity, ATP content and NADPH/NADP ratio were found to be decreased significantly in A549 cells, while ROS content was distinctly increased (Figure 1D-F). In contrast, although the LDH enzyme was also inhibited in HBE cells in a dose-dependent way, however, the glucose metabolism was less influenced (Figure 1G-I).

### Cycle arrest induced by LDH-A inhibition in NSCLC cells

Cell cycle is a reflection of cell growth and division, which is easily disturbed by external stress [26]. Therefore, we next examined the effects of LDH-A inhibition on cycle progression in HBE, H1395 and A549 cells. All the cells were treated with different concentrations of oxamate (0, 20, 50, 100mmol) for 24h, and then were analyzed by flow cytometry after PI staining.

**Figure 2:**
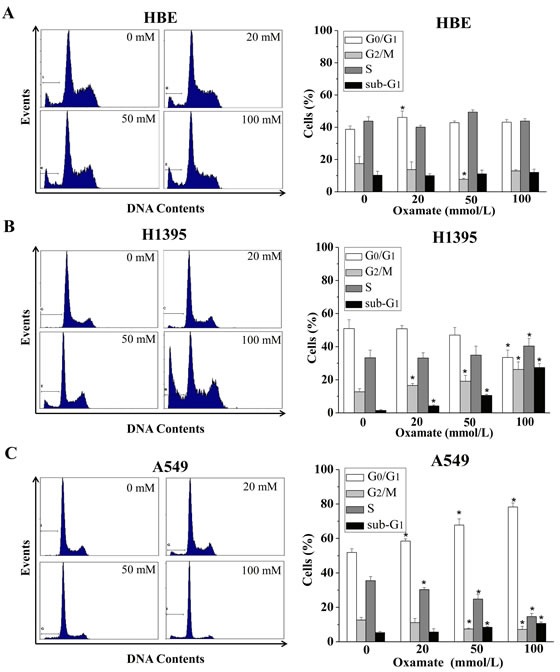
Different effects of oxamate on the cell cycle arrest in NSCLC cells and normal lung epithelial cells Cells were treated with 0, 20, 50, 100 mM oxamate for 24h, and then were analyzed by flow cytometry after PI staining. (A) HBE cells; (B) H1395 cells; (C) A549 cells. Each phase of cell cycle (sub-G_1_, G_0_/G_1_, G_2_/M, S) in oxamate-treated cells was compared with the respective phases in untreated cells, All Data above represented the average of three independent experiments and were shown as means ± SD, * p<0.05 versus control.

**Figure 3:**
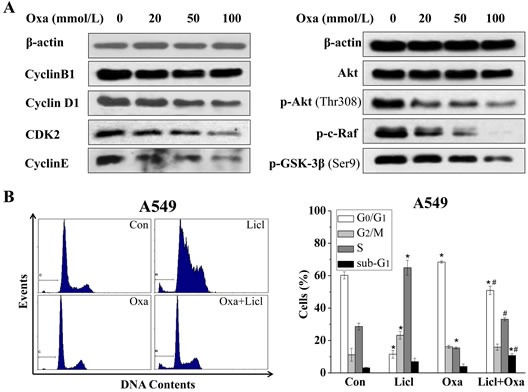
G_0_/G_1_ arrest in A549 cells is mediated by Akt-GSK-3β-cyclinD1 signal pathway (A) Cells were treated with increasing concentrations of oxamate for 24h, after treatment, cells were harvested, lysed, and then analyzed by western blotting with the indicated antibodies, β-actin was used as a loading control. (B) A549 cells were pre-treated with 20 mM LiCl followed by combination treatment with 100 mM oxamate for 24 h, then prepared for flow cytometry analysis after PI staining.

As shown in Figure 2A, no dose-dependent changes were observed in cell cycle progression with the concentrations of oxamate in normal lung epithelial HBE cells, in accordance with the effect on the cell viability. Meanwhile, we found that the numbers of H1395 cells in G_2_/M phase were increased in a dose-dependent manner after treatment with 0, 20, 50, 100mmol oxamate, the proportions of cells at G_2_/M were 12.74±1.78%, 16.55±1.20%, 19.09±3.56%, and 26.20±4.57%, respectively (p<0.05), while the percentages of cells in G_0_/G_1_ phase were consequently deceased (Figure 2B). Intriguingly, there was another scenario in A549 cells, as shown in Figure 3C, oxamate induced significant G_0_/G_1_ arrest in A549 cells, after being exposed to 0, 20, 50, 100 mmol/L oxamate, the proportions of cells at G_0_/G_1_ phase were 51.88±0.14%, 58.50±0.28%, 67.79±0.58% and 78.24±2.4%, respectively (p<0.05). In addition, we noticed that the ratios of cells in the sub-G_1_ phase were not elevated significantly except in H1395 cells.

### G_0_/G_1_ arrest in A549 cells was dependent on the activation of GSK-3β

**Figure 4:**
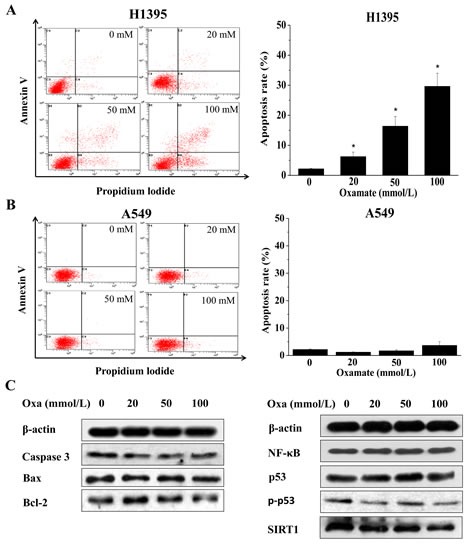
LDH-A inhibition by oxamate induces apoptosis in H1395 while not in A549 cells Cells were treated with 0, 20, 50, 100 mM oxamate for 24h, the apoptosis rates were tested by AnnexinV/PI staining. (A) A549 cells; (B) H1395 cells. (C)The proteins involved in apoptosis as well as their upstream regulators were analyzed by western blotting in A549 cells, after treatment with oxamate, β-actin was used as a loading control.

Earlier studies have reported that G_2_/M arrest was induced by the inhibition of LDH-A or other enzymes involved in glycolysis in cancer cells [18, 19, 27, 28]. However, we found that oxamate induced significant G_0_/G_1_ arrest, instead of G_2_/M arrest in A549 cells in our study. To explore the molecular mechanisms under the G_0_/G_1_ arrest in A549 cells, we performed western blotting to detect the changes of cell cycle-related proteins in A549 cells after treatment of oxamate for 24h. As shown in Figure 3A, we found that the expression of cyclin D1 was in a dose-dependent decrease, accompanied by a reduction in the levels of CDK2 and cyclin E, while the expression pattern of cyclin B1 was basically unchanged (Figure 3A). Next, we continued to test other proteins in upstream signal pathways affecting cell cycle, the results showed that the expressions of p53, SIRT1, NF-κB did not alter significantly after treatment of oxamate (Figure 4A). However, the PI3K-Akt signaling pathway, which plays an important role in cell proliferation, metabolism and cycle regulation [29], was obviously inhibited. Notably, the levels of p-Akt and p-c-raf were decreased dramatically in a dose-dependent way. Consistent with this, the phosphorylation of glycogen synthase kinase-3β (GSK-3β) at Ser-9, a substrate of p-Akt [30, 31], was also inhibited significantly, accompanied with an enhanced function.

It has been long known that cyclin D1 can be down-regulated by the activation of GSK-3β, which phosphorylated cyclin D1 that triggers its subsequent degradation [32, 33]. To further verify the role of Akt-GSK-3β-cyclin D1 signal pathway in the G_0_/G_1_ arrest induced by LDH-A inhibition, we therefore employed lithium chloride (LiCl, 20 mmol/L), an inhibitor of GSK-3β, to treat A549 cells with oxamate together. The results, in accord with our expectations, showed that the G_0_/G_1_ arrest induced by oxamate was reversed almost completely by lithium (Figure 3B). Taken together, these data indicated that oxamate-induced G_0_/G_1_ arrest was possibly mediated by the changes of G_0_/G_1_ cyclins, especially caused by the degradation of cyclin D1, which resulted from the de-phosphorylation and activation of GSK-3β.

### Oxamate triggered apoptosis in H1395 while not in A549cells

Apoptosis is one of the major responses to LDH-A inhibition in cancer cells, as previous studies reported [13-15]. To investigate the effect of oxamate on the apoptosis in different NSCLC cells, AnnexinV/PI double staining assays were conducted. As shown in Figure 4A, after treatment with 0, 20, 50, 100 mmol/L oxamate for 24h, the percentages of cells at apoptosis increased significantly in H1395 cells, the apoptosis rates were 2.15±0.16%, 6.24±1.50%, 16.36±3.23% and 29.66±4.34% respectively. By contrast, the apoptosis rates in A549 cells after treatment with oxamate were all at low levels, similar with the control group.

The mechanism under the apoptosis induced by LDH-A inhibition has been well studied in other similar studies, and the results indicate that ROS mediated mitochondrial pathway plays a key role [13, 15, 17]. In the present study, the ROS contents were also enhanced in A549 cells, but relatively lower than the extent in other cells (Figure 1F). To verify that there was no apoptosis induced by oxamate in A549 cells, the expression changes of apoptotic markers, including bcl-2, bax and caspase-3, were explored by western blotting, the results showed that these proteins did not change clearly after treatment with oxamate. Negative results were also obtained with other key regulators in apoptosis, such as p53, SIRT1, NF-κ B (Figure 4C).

### Oxamate induced protective autophagy in A549 cells

**Figure 5:**
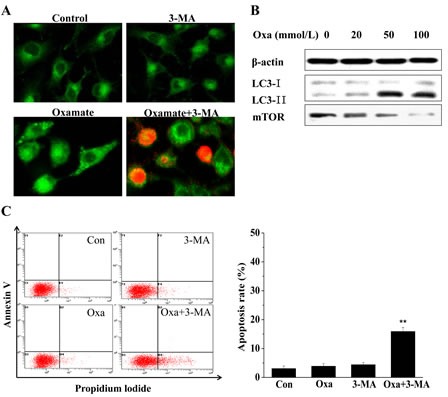
Oxamate induced protective autophagy in A549 cells (A) A549 cells were exposed to 100mM oxamate with or without 10mM 3-MA, then were observed under a fluorescence microscope after MDC/PI dual staining, green fluorescence represents autophagic vacuoles whereas red fluorescence represents apoptotic cells. (B) Western blotting was employed to detect the expression levels of LC3 and mTOR, two autophagy-related proteins. β-actin was used as a loading control. (C) The apoptosis rates were analyzed by quantitative flow cytometry. Three independent experiments were repeated and the results were shown as means ± SD, * p<0.05 versus control.

In our previous experiments, LDH-A inhibition by oxamate was found not induce apoptosis in A549 cells, we tried to explore the mechanism of growth suppression in A549 cells using MDC staining. After treating A549 cells with oxamate for 24h, the appearance of acidic vesicles was indeed observed in the cytoplasm of oxamate-treated cells, indicating that autophagy might occur in A549 cells. Next, we detected the expression changes of LC-3, an autophagy marker, to confirm the phenomenon. As shown in Figure 5B, the expression of LC3-II protein was increased in A549 cells following 24 h of oxamate treatment, especially in 50 and 100 mM groups, whereas the expression level of LC3-I was decreased to some extent, which is compatible with an enhanced autophagic flux.

To further investigate the role of autophagy in the anti-proliferation of oxamate in A549 cells and its relationship with apoptosis, we next adopted 3-methylademine (3-MA, 10 mM), an autophagy inhibitor, to treat A549 cells simultaneously with oxamate for 24h, and MDC/PI dual staining showed that there were a large number of PI-positive cells in 3-MA combined with oxamate group. Consist with the results of MDC/PI staining, the quantitative flow cytometry demonstrated that the apoptosis rate was significantly higher in the cells treated with both 3-MA and oxamate, compared to the control cells or the cells treated with oxamate alone. Therefore, the results suggested that LDH-A inhibition by oxamate induced autophagy in A549 cells, which protected the cells against apoptosis.

## DISCUSSION

LDH-A has been widely investigated as an anti-tumor target in previous studies, and accumulating evidence indicates that inhibition of LDH-A induces apoptosis though mitochondrial pathway mediated by oxidative stress [13, 15, 17, 18]. To date, it's still unkown that whether autophagy can be induced by LDH-A inhibition. In the present study, we firstly revealed that oxamate, a classic LDHA inhibitor induced autophagy in A549 cells, and addition of autophagy inhibitor 3-MA with oxamate caused apoptosis again in A549 cells. Another interesting phenomenon found in our study was that LDH-A inhibition induced G_0_/G_1_ arrest dependent on the activation of glycogen synthase kinase-3β. Most of all, these findings were based on a multiple context, we employed HBE cells as a normal control, and also observed the apoptosis and G_2_/M arrest induced by LDH-A inhibition in H1395 cells. The distinct responses to oxamate in different NSCLC cells suggest that the effects of LDH-A inhibition are more complicated than they appeared before.

LDH-A is a key enzyme in anaerobic glycolysis, by catalyzing the conversion of pyruvate into lactate. When the LDH-A is inhibited, more pyruvate will enter into the tricarboxylic acid (TCA) cycle and more oxygen is needed. However, since cancer cells tend to overly depend on aerobic glycolysis, which generates ATP faster and provides more precursors to meet the metabolic requirements of rapid proliferation [34], TCA cycle and subsequent mitochondrial oxidative phosphorylation (OXPHOS) pathway in them are always impaired and dysfunctional [35]. Consequently, increased ROS are produced, which activate apoptosis though caspase-3 pathway [36]. In the present study, we further confirmed the apoptosis induced by LDH-A inhibition in H1395 cells, and also found that LDH-A inhibition induced G_2_/M arrest, as reported previously [18, 27, 28]. Moreover, the toxicity of oxamate was much lower in normal lung epithelial HBE cells, the results demonstrate that LDH-A is also a potential target for cancer therapy in NSCLC patients.

Autophagy is a self-eating process, in which cellular components are degraded through the lysosomal system to maintain homeostasis [37]. Under metabolic stress, cancer cells can use autophagy as a source of energy and biomolecules to adapt and survive in the unfavorable environment [38]. Here, we firstly reported that LDH-A inhibition induced autophagy in human non-small cell lung cancer A549 cells, as evidenced by appearance of autophagic vacuoles and LC-3 degradation, our data also showed that the Akt-mTOR signaling pathway was inhibited significantly after oxamate treatment, and might involve in the regulation of the metabolism and autophagy induced by LDH-A inhibition. Moreover, the autophagy inhibitor 3-MA blocked the autophagy and triggered apoptosis again in human A549 cells, which was similar to the situation in H1395 cells, suggesting that the autophagy was a protective mechanism against apoptosis and contributed to the drug resistance of LDH-A inhibitors. Recent studies have shown that autophagy plays an important role in glucose metabolism and the maintenance of lung tumor's malignancy [39]. Basing on these above, our study further indicates that combined inhibition of autophagy is a worthy strategy to overcome the drug resistance of LDH-A inhibitor in NSCLC cells.

Another interesting phenomenon is that we observed different cell cycle arrest coupled with apoptosis or autophagy. In H1395 cells, after treatment with oxamate, cells arrested in G_2_/M cycle were associated with damaged mitochondrial function and susceptive to apoptosis, however, G_0_/G_1_ quiescence in A549 cells was related to increased autophagy activity, which allows cells to survive under the metabolic stress. Especially, our study further found that the G_0_/G_1_ arrest in A549 cells was dependent on the activation of p-GSK-3β, which is at the down-stream signal transduction of Akt/mTOR pathway [40]. In agreement with our results, An, *et al* currently reported that autophagy is necessary for G_0_/G_1_ arrest under nitrogen starvation in saccharomyces cerevisiae, and concluded that such cycle arrest might permit the cells to adapt the nutrient deprivation [41]. In addition to this, our results also demonstrated that when the oxamate-induced G_0_/G_1_ quiescence was disrupted by lithium, the changes in the percentage of apoptotic cells were not significant, the results indicate that G_0_/G_1_ arrest might be an accompaniment activity with autophagy, however, the intervention of cycle progression will not determine the final destiny of cells with LDH-A inhibition.

Since lung cancer is one kind of highly heterogenous tumors, biomarkers are vitally important in improving the effectivity of target therapy [42]. As is well-known, EGFR mutation has been proven successfully as a predictor in TKIs (tyrosine kinase inhibitors), which save many patients' lives as well as money [43]. As the development of more effective LDH-A inhibitors (also including other glycolysis inhibitors), now there is a pressing need to seek for biomarkers to predict sensitivity and screen patients who will benefit most from those inhibitors [19, 44, 45]. For instance, not long ago, Birsoy *et,al* reported that mtDNA mutations might be useful in determining the sensitivity of cancer cells to glucose limitation [46]. Our results indicated that the biological consequences of LDH-A inhibition are more complex than we thought before in NSCLC cells, and the signal molecules in Akt/mTOR and autophagy pathway might be of potential value to predict the efficacy of LDH-A inhibitors.

In conclusion, we find that NSCLC cells exhibit different responses to LDH-A inhibition in our study, and provide novel insights into the signaling pathways shifting cancer cells towards apoptosis or autophagy, as well as different cell cycle arrests, which are helpful for searching biomarkers to monitor the efficacy of glycolysis inhibitors and contribute to more favorable outcomes in the future clinical trials. The results also suggest that combined autophagy inhibition may be an attractive strategy to enhance the sensitivity of LDH-A inhibitors in drug-resistant cells.

## MATERIALS AND METHODS

### Reagents and cell culture

Oxamate sodium was purchased from Sigma-Aldrich Corp (St. Louis, MO, USA). Human non-small cell lung cancer cell lines including A549, H1975 and H1395 were used, normal lung epithelial cell line HBE was employed as a normal control. All the cell lines were obtained from the American Type Culture Collection (ATCC, Manassas, USA), and cultured in Dulbecco's modified Eagle's medium (DMEM, Gibco) containing 10% fetal bovine serum at 37 °C under 5% CO_2_.

### MTT assay

MTT (methye thiazolye telrazlium) assay was used to test the effects of oxamate sodium on cell viability at different concentrations or times. Cells were seeded at 10^4^/well in 96-well plates, and treated with fresh media containing different doses of oxamate (0-100 mmol/L). After 24h, 48h and 72h incubation, respectively, 20 μl of MTT solution (5 mg/L) was added into each well, then the plates were incubated in the dark for 4 h. The supernatant was removed and the precipitates were dissolved in 150 μl dimethyl sulfoxide for 10 min. Optical density was measured using a microplate reader (Bio-Tek Instruments, Inc., Winooski, VT, USA) at 570 nm.

### LDH activity test

LDH Activity Assay kit (Biovision, Tucson, AZ, USA) was used to determine the intracellular LDH activity. In this test, LDH reduces NAD to NADH, which interacts with a specific probe to produce a color (λ_max_ = 450nm), which is then detected by colorimetric assay. Results were expressed as percentage of LDHA activity normalized to protein concentration, which were measured by BCA protein assay kit (Beyotime, Haimen, China).

### ATP detection

Intracellular ATP was detected using a luciferase-based ATP assay kit (Beyotime, Haimen, China), according to the manufacturer's instructions. Cells treated with different doses of oxamate were harvested and lysed. Then, optical density was measured using a microplate reader (Bio-Tek Instruments, Inc.) and normalized to protein concentration.

### Reactive Oxygen Species Measurements

Cells were stained with 2′,7′-dichloro-fluoresce in diacetate (DCFH-DA) according to the manufacturer's instructions (Beyotime, China), and the fluorescence intensity was measured by a flow cytometry at 530nm.

### Analysis of cell cycle distribution

After 24h incubation with different doses of oxamate, cells were collected and fixed with 70% pre-cooled ethanol overnight, after staining with propidium iodide (10 μg/ml; Sigma-Aldrich) in the dark for 30 min. Flow cytometry was performed on the FACS Calibur system (Becton Dickinson, San Jose, CA, USA) and cell cycle distribution was analyzed by means of ModFit LT software (Becton Dickinson, CA, USA).

### Apoptosis analysis

Annexinv-FITC apoptosis kit (BD Biosciences, San Jose, CA, USA) was employed to test apoptosis. Cells were harvested after 48h oxamate treatment, then stained with Annexinv/PI for 30 min. The results were analyzed by the FACSCalibur system with ModFit's LT software.

### Western blot

Protein was extracted using IP lysing buffer (Beyotime, China), and was quantified by a BCA Protein Assay Kit (Beyotime, China). 50 ug protein lysates were separated on SDS/PAGE gel, and transferred onto PVDF membrane (Millipore), then immunoblotted with primary antibodies. All primary antibodies were diluted 1:2000 in TBS containing 5% nonfat milk.

### MDC/PI dual staining

MDC (monodansylcadaverin) is a fluorescent compound that can detect autophagic vacuoles in cells, and PI (Propidium iodide) is a marker of cell death. To observe the phenomenon of cell autophagy and death at the same time, MDC/PI dual staining was employed in our study. After pretreatment with oxamate for 24h, cells were stained with 0.05mmol/L MDC and 50μg/ml PI at 37°C for 30min, then were observed under a fluorescence microscope at excitation wavelengths of 350nm and 630nm.

### Statistical analysis

All Data were obtained from at least three independent experiments and were shown as means ± SD, two-tailed Student's t-test was used to assess the difference between two groups, p<0.05 was considered to be statistically significant. All of the statistical analyses were carried out using SPSS version 17.0.
